# Viruses and the host replisome: discovering oncogenic mechanisms of small DNA tumor viruses

**DOI:** 10.1128/jvi.01691-25

**Published:** 2026-01-07

**Authors:** Christopher D. Collins, Matthew Stefely, Kavi Prem Milan Mehta, Megan E. Spurgeon

**Affiliations:** 1 John W. and Jeanne M. Rowe Center for Research in Virology, Madison, Wisconsin, USA; 2 Morgridge Institute for Research 145254, Madison, Wisconsin, USA; 3 Department of Oncology, McArdle Laboratory for Cancer Research, University of Wisconsin-Madison School of Medicine and Public Health 5229 , Madison, Wisconsin, USA; 4 Department of Comparative Biosciences, University of Wisconsin-Madison School of Veterinary Medicine 5229 , Madison, Wisconsin, USA; College of Agriculture & Life Sciences, University of Arizona, Tucson, Arizona, USA

**Keywords:** DNA tumor viruses, human papillomavirus, Merkel cell polyomavirus, DNA replication, DNA damage repair, host replisome, DNA damage, iPOND, viral oncoproteins

## Abstract

Human papillomavirus (HPV) and Merkel cell polyomavirus (MCPyV) are DNA tumor viruses that cause human cancer. The mechanisms by which HPV and MCPyV oncoproteins induce genomic instability are not well defined. This minireview discusses the influence of these oncoproteins on the repertoire of proteins at replicating DNA, known as the host replisome, and discusses how new technologies like isolation of proteins on nascent DNA (iPOND) can drive the discovery of viral dysregulation of the host replisome to enhance our understanding of viral oncogenesis.

## INTRODUCTION

Tumor viruses, or viruses that are etiological agents of tumorigenesis and/or cancer, were first discovered in the early 20th century when Peyton Rous isolated a “filterable agent” from chicken sarcomas that would later be named Rous sarcoma virus (1). In the 1930s, Robert Shope discovered the first mammalian tumor virus that caused papillomas in cottontail rabbits that were later shown to undergo malignant progression when treated with co-carcinogens. Anthony Epstein and Yvonne Barr identified the first human tumor virus in the 1960s, Epstein-Barr virus, isolated from Burkitt’s lymphoma tumors, representing the first connection between a virus and human cancer. We now know that several other viruses, including human papillomavirus (HPV), Human T-lymphotropic virus 1, Kaposi’s sarcoma-associated herpesvirus, hepatitis B and C viruses, and Merkel cell polyomavirus (MCPyV), can cause human cancers (2). Together, these seven RNA and DNA human tumor viruses contribute to at least 15% of the global cancer burden (3, 4).

HPV and MCPyV are both small DNA tumor viruses. HPVs with oncogenic potential, also known as high-risk HPVs, account for at least 5% of all human cancers worldwide (5). MCPyV, on the other hand, is the most recently discovered human tumor virus and is only known to cause the rare cancer type Merkel cell carcinoma (MCC) (6). However, these two tumor viruses share similar oncogenic mechanisms. In this review, we will discuss the current state of knowledge and future perspectives regarding how HPV and MCPyV, both DNA tumor viruses, dysregulate the host replisome as a potentially common means by which they cause human cancer.

## HPV AND MCPyV: DNA TUMOR VIRUSES AND AGENTS OF MALIGNANCY

HPV is the most common sexually transmitted disease in the world (7). The CDC estimates that nearly all sexually active individuals will become infected with HPV at some point in their lifetime, and an estimated 13 million new cases occur annually (8, 9). High-risk HPVs are the etiological source of more than 99% of all cervical cancer cases and contribute significantly to new cases of anal, oropharyngeal, and penile cancers, while low-risk types can result in benign genital warts and respiratory papillomatosis (5, 10). Most HPV infections are cleared, but persistent infection with high-risk HPV types such as HPV16 or HPV31 can cause severe malignancies. Despite advances in screening and availability of prophylactic vaccines, HPV remains the most oncogenic tumor virus among known human tumor viruses (10). These statistics, together with the fact that there are currently no treatments or cures for HPV infections, highlight the importance of further characterizing host-HPV interactions and the mechanisms of HPV-mediated carcinogenesis.

Currently, more than 200 distinct HPV types have been identified, including alpha and beta types that are most frequently associated with human cancers (11). Beta HPV types exhibit tropism for cutaneous epithelia, and high-risk types can facilitate carcinogenesis of UV-associated skin carcinomas (12–14). High-risk alpha HPVs exhibit mucosal tropism, with types HPV16, HPV18, and HPV31 being most frequently associated with human cancers (15). Both alpha and beta HPVs also include low-risk types associated with benign neoplasms (16).

MCPyV was discovered in 2008 and is currently the only human polyomavirus known to cause human cancer (17). MCPyV is linked to MCC, a rare but extremely aggressive form of neuroendocrine carcinoma of the skin. The incidence of MCC has tripled over the last 40 years, with a 95% increase in the US between 2000 and 2013 (18, 19), and the number of new cases is projected to increase by 32%–40% by the end of 2025 (20), likely due to an increasing aging population. MCC forms at several anatomical sites, although tumors tend to form on sun-exposed skin and are more prevalent in elderly, light-skinned, and/or immunosuppressed populations (18, 19, 21–24). MCC is so named due to its phenotypical resemblance to Merkel cells (25), also known as “touch cells,” which are localized to the basal layer of the epidermis where they tend to cluster around hair follicles and transduce mechanical stimuli to sensory neurons through β-adrenergic receptors (26–28).

Portions of the MCPyV viral genome have been detected in ~80% of (MCC) tumors, with most of these tumors harboring clonally integrated viral DNA (17), indicating that viral genome integration precedes clonal expansion of tumor cells and likely helps drive carcinogenesis. The remaining 20% of virus-negative MCC cases tend to occur more often in sun-exposed regions of skin and exhibit a high mutational burden predominated by UV-induced mutations (29–31). Mutations in MCPyV-negative MCC tumors frequently occur in *RB1* and *TP53* genes with adverse effects on tumor suppressor function (30, 32), activities that are similarly dysregulated by the MCPyV tumor antigens in virus-positive cases. While there are various MCC-related isolates of MCPyV containing tumor-specific truncations in large T antigen (LT) (33), it is not currently known whether there are multiple viral types as found with HPV.

## HPV AND MCPyV: TROPISM, GENOME ORGANIZATION, AND REPLICATION

HPV and MCPyV viruses share several similarities but also have key differences. Both viruses are common and generally considered part of the natural human flora. HPV exhibits tropism for the stratified epithelium, with distinct preferences for cutaneous or anogenital and oral mucosal tissues in a type-specific manner (13). HPV specifically infects basal epithelial cells of the stratified squamous epithelium, and the viral life cycle is inextricably linked to terminal differentiation, which prompts viral genome amplification and a switch to expression of the late structural genes L1 and L2 in preparation for progeny virion production (34). MCPyV is also ubiquitous in the population and appears to exhibit a general tropism for skin (35–39). Several lines of evidence from *in vitro*, *ex vivo*, and organoid experiments indicate that dermal fibroblasts support the full MCPyV life cycle (37, 40), although whether this cell type gives rise to MCPyV-positive MCC remains unclear.

Both HPV and MCPyV are nonenveloped viruses with double-stranded DNA genomes. The HPV and MCPyV viral genomes are approximately 8kb and 5.4kb, respectively, and both are partitioned into early and late regions separated by either a long control region, also known as the upstream regulatory region (LCR/URR) in HPV or a non-coding control region (NCCR) in MCPyV, which contains origins of replication. The HPV genome has two major promoters, the first “early” being located within the LCR and a second “late” promoter contained within the E7 open reading frame (ORF). These promoters are unidirectional, and genes are bi-cistronically or polycistronically transcribed into a single pre-mRNA that undergoes “leaky scanning” translation and extensive splicing to generate distinct gene products (41). The HPV early region encodes ORFs for regulatory proteins E1, E2, E4, E5, E6, and E7 that play a variety of roles in regulating productive viral replication and gene expression, as well as cell cycle regulation and apoptotic signaling (42). Replication of the viral episome is initiated through helicase activity of E1 and transcriptional regulation performed by E2, which also plays an essential role in viral replication by recruiting E1 to origins of replication and stabilizing those interactions (43, 44). The MCPyV NCCR, on the other hand, contains bidirectional promoter/enhancer elements that regulate early and late gene expression over the course of infection (45). Expression of the MCPyV early region occurs early during infection and is driven by a single promoter, with this locus encoding multiple viral proteins, termed tumor (T) antigens, that are produced through alternative splicing and that are required for viral replication and amplification. MCPyV LT contains an origin-binding domain and functions as a viral helicase to potentiate viral DNA replication, and these activities are supported by the MCPyV small T antigen (ST) (45–47). MCPyV also expresses a 57 kT protein, whose function remains unclear, as well as a protein called alternative LT open reading frame (ALTO) that regulates the host immune response and promotes viral persistence (48–51). Therefore, multiple HPV and MCPyV viral proteins play essential roles in viral replication, pathogenesis, and ultimately cancer development.

## VIRAL ONCOPROTEINS ARE DRIVERS OF IMMORTALIZATION, CELLULAR TRANSFORMATION, AND CARCINOGENESIS

During HPV-induced and MCPyV-induced carcinogenesis, portions of the viral genome are often integrated into the host genome (17, 52). These integration events render the virus replication incompetent, as important regulatory functions present in viral genes are lost. In the case of HPV, viral genome integration leads to the loss of the viral E1 helicase and E2 protein, which normally represses expression of the E6 and E7 oncogenes (52, 53). Therefore, viral genome integration not only leads to loss of the viral helicase necessary for viral replication but also results in increased expression and stability of E6 and E7 transcripts, creating a cellular growth advantage (54, 55). In the case of MCPyV, MCC tumors contain integrated portions of the viral early region and tumor-specific truncations in the C-terminus of the LT helicase protein (33). Integration is generally a dead end for viral replication due to the loss of viral genes that encode key regulatory and structural proteins.

Viral oncoproteins are characterized by their ability to drive uncontrolled cell proliferation and survival, resulting in carcinogenesis through persistent and prolonged expression (56). The process of virus-induced host cell transformation is dependent on virus-host interactions that create a cellular environment conducive to cellular proliferation and virus replication. Viral oncoproteins support these conditions through a variety of mechanisms, including most notably the inactivation of tumor suppressor pathways, but also the induction of replication stress and impairment of DNA damage response (DDR) pathways that increase genomic instability, activation of oncogenes, disruption of cell cycle regulation, immune evasion, and chronic inflammation (57).

HPV-induced oncogenesis involves two viral proteins, E6 and E7, which, through virus-host interactions, dysregulate tumor suppressor pathways in infected cells, induce S phase to facilitate productive viral replication, and potentiate several key hallmarks of cancer, including sustained proliferative signaling and resistance to cell death (58). HPV E6 and E7 are among the first genes to be expressed during the viral life cycle. During viral replication, these oncoproteins also contribute to genomic instability through dysregulation of several key host DDR factors (59–64). The mechanisms used by high-risk HPV E6 and E7 to induce aberrant cell cycling and genomic instability are multimodal but are initiated by their increased affinity for the tumor suppressors p53 and pRb, respectively, to prevent apoptosis and promote S phase cell cycle entry compared to low-risk HPV types (65, 66). The HPV oncoproteins are highly multifunctional and widely influence various cellular processes during infection and pathogenesis (58).

MCPyV-induced carcinogenesis is potentiated by the functions of its viral oncoproteins, LT and ST. In a manner strikingly similar to high-risk HPV E7 oncoproteins, MCPyV LT interacts with and inactivates the tumor suppressor pRb through a conserved LxCxE motif, releasing E2F for nuclear translocation with subsequent upregulation of E2F targets involved in cellular proliferation, metabolism, and enhanced cellular progression from G1 to S phase, optimizing conditions for viral replication (33, 67). This oncogenic effect is preserved through retention of the pRb-binding domain in truncated forms of LT found in MCC (33). MCPyV also evades p53-induced growth arrest through interactions between ST and the chromatin remodeling EP400 complex that upregulate expression of the E3 ubiquitin ligase and regulator of p53 protein stability MDM2, effectively inactivating p53 signaling and enabling infected cells to bypass the G1/S checkpoint critical for regulating proliferation and maintaining genomic integrity (68, 69). The ST oncoprotein also regulates protein synthesis by mediating key signaling components of the mammalian target of rapamycin pathway, a function that underlies its *in vitro* transformation capacity (70). Given the paucity of mutations in MCPyV-positive MCC tumors (30), the functions of the LT and ST proteins are believed to be the prominent drivers of MCC carcinogenesis.

## VIRAL DYSREGULATION OF HOST DDR PATHWAYS

The DDR is an important cellular process that supports genomic integrity and consists of a complex network of pathways that recognize and transduce stress response signals, resulting in cell cycle regulation, activation of damage-specific repair pathways, or apoptosis if damage is not repaired (71). Three master regulatory kinases that activate DDR downstream effector signaling are ATM, ATR, and DNA-PKcs. ATM and DNA-PKcs respond to double-strand breaks (DSBs), while ATR responds to the presence of single-stranded DNA. DNA DSBs in particular pose an urgent and significant obstacle to faithful DNA replication due to potential loss of templated genetic information and risk of chromosomal rearrangements. The MRN (Mre11-Rad50-Nbs1) complex is one important DDR complex that initiates ATM-dependent signaling to repair the damage through homologous recombination (HR). Competent HR also requires proficiency of the BRCA family of proteins to load RAD51 and form nucleoprotein filaments on resected ssDNA that facilitate invasion of the homologous strand. While HR provides a high-fidelity mode of repair, template availability is restricted to S and G2 phases of the cell cycle. Due to this restriction, cells can also employ a more error-prone mechanism of repair, nonhomologous end-joining (NHEJ), initiated by recognition of DSBs by the Ku complex (Ku70-Ku80-DNA-PKcs). NHEJ can result in insertion or deletion events, or even chromosomal translocations that permit DNA replication and cell cycling, but at the cost of increased genomic instability.  

While it is generally accepted that viruses co-opt these mechanisms to preserve the integrity of their own genome and potentiate replication, studies demonstrate that specific host DDR pathways activated by HPV are required for viral genome amplification and copy number maintenance (59, 60, 62, 72–75). Although it was determined for simian virus 40 (SV40) that ATM is required for resolving concatemer formation during rolling-circle replication (76), no evidence indicates this is the case in HPV, despite HPV utilizing either rolling-circle (77) or recombination-based replication for amplification (78, 79). Several lines of evidence show further HPV-induced dysregulation of host DDR pathways, HR, and NHEJ, to name only a few. Expression of HPV E6 and E7, independently or together, results in increased DSBs, activation of multiple DDR and replication stress response pathways, and depletion of free nucleotide pools, the building blocks of replication (60, 80). E6 additionally stabilizes c-Myc, which increases transcriptional hTERT activation and prevents telomere length shortening. E6 and E7 singly or co-expressed disrupt HR signaling, and E7 hijacks RNF168, disrupting NHEJ (61, 81–87).

HPV genome amplification during the productive phase of its life cycle relies on constitutive activation of ATR (64, 88–90) and ATM signaling and induces phosphorylation of several downstream ATM effectors, including CHK2, H2AX, 53BP1, NBS1, SMC1, and BRCA1. SMC1 is also required for HPV31 episomal maintenance through interaction with virally encoded CTCF-binding sites (74). In keratinocytes stably maintaining HPV31 episomes, viral replication occurs in nuclear replication centers, or foci, that colocalize with ATM components, CHK2, FANCD2, and γ-H2AX (63, 91). HPV31 has also been shown to recruit DNA repair proteins to these nuclear foci, such as the NHEJ mediator 53BP1, and HR repair factors RAD51 and BRCA1 are important for viral replication (63). The preferential recruitment of HR machinery to viral DNA is exacerbated by replicative stress, such as nucleotide depletion, and occurs at the expense of host DNA repair, coinciding with a higher incidence of DSBs within chromosomal DNA. The recruitment of RAD51 and BRCA1 in particular is reliant upon expression of HPV oncoproteins E6 and E7 and can occur independently of viral replication (59, 60); however, little is currently understood about how these oncoproteins dysregulate HR once viral integration has occurred, nor the direct mechanisms by which they activate these responses and induce genomic instability.

Given the smaller body of research on MCPyV, our understanding of how this virus interacts with host DDR factors is still developing. However, the replication stress response and replisome disruption are important to other polyomaviruses. Related and better-characterized polyomavirus proteins, such as SV40 LT, dysregulate ATM, a function important for viral replication (92, 93). As further evidence of polyomavirus dependence on host DDR factors, SV40 viral replication is significantly decreased in ATM-deficient, p53-null MEFs (mouse embryonic fibroblasts), and in MEFs expressing phosphorylation-defective SMC1 (94). Notably, expression of SV40 LT alone in BJ/tert fibroblasts is sufficient to induce replication stress, evidenced by focal accumulation of γ-H2AX and 53BP1, with downstream activation of FANCD2 (95). DDR also enhances human JC polyomavirus replication (96). Given that dysregulation has been extensively characterized in several other members of the *Polyomaviridae* family, it is possible that this may be a common mechanism shared with MCPyV.

Indeed, evidence continues to emerge that MCPyV, like other polyomaviruses, provokes replication stress and DNA damage signaling. MCPyV infection increases both ATM and ATR signaling, resulting in an accumulation of DDR factors at viral replication centers that facilitate viral replication (97, 98). Notably, both MCPyV LT and ST oncoproteins can induce genome instability in the absence of viral replication. LT alone is sufficient to activate DNA damage signaling pathways and is itself a target of phosphorylation by ATM (97, 99). ST expressed alone also modulates host DDR as evidenced by the accumulation of γ-H2AX and activation of ATM, CHK2, and 53BP1 (100). A recent study found that the MCC-specific truncated LT alone increases DDR protein expression and identified several DDR proteins as interactors, including the histone lysine methyltransferase EHMT2, which may be involved in regulating the replication stress response in MCC cells (101). Through its manipulation of E3 ubiquitin ligases, ST expression causes several chromosomal and mitotic spindle abnormalities, such as aneuploidy, chromosomal breaks, and micronuclei (102). ST also binds to and redirects the activity of various host transcriptional regulators and chromatin remodeling factors to regulate host and viral transcription (68, 103). Furthermore, in multiple, independent MCPyV transgenic murine models, expression of truncated LT and/or ST in the skin results in γ-H2AX foci and ATM activation (104, 105). The effects of MCPyV on genome stability are particularly interesting when one considers that MCPyV-positive MCCs exhibit characteristics of neuroendocrine cells (i.e., Merkel cells), perhaps indicative of cellular reprogramming of the originally infected cell. Recent studies suggest that aberrant DDR signaling can result in substantial cellular rewiring through signal transduction cascades that can involve cytoplasmic processes (106). Future studies promise to yield further knowledge on the role of DDR and maintenance of genome stability in MCPyV viral replication, persistence, and MCC development.

## THE REPLISOME AND IMPLICATIONS FOR GENOMIC INSTABILITY AND CANCER

DNA replication is intimately linked to robust, redundant, and effective DNA repair mechanisms that evolved to respond to multiple DNA lesions and structures to ensure maintenance of genome stability. A high proportion of cancers have defects in DNA repair genes, with at least 5%–10% being germline mutations (107, 108).

**Fig 1:**
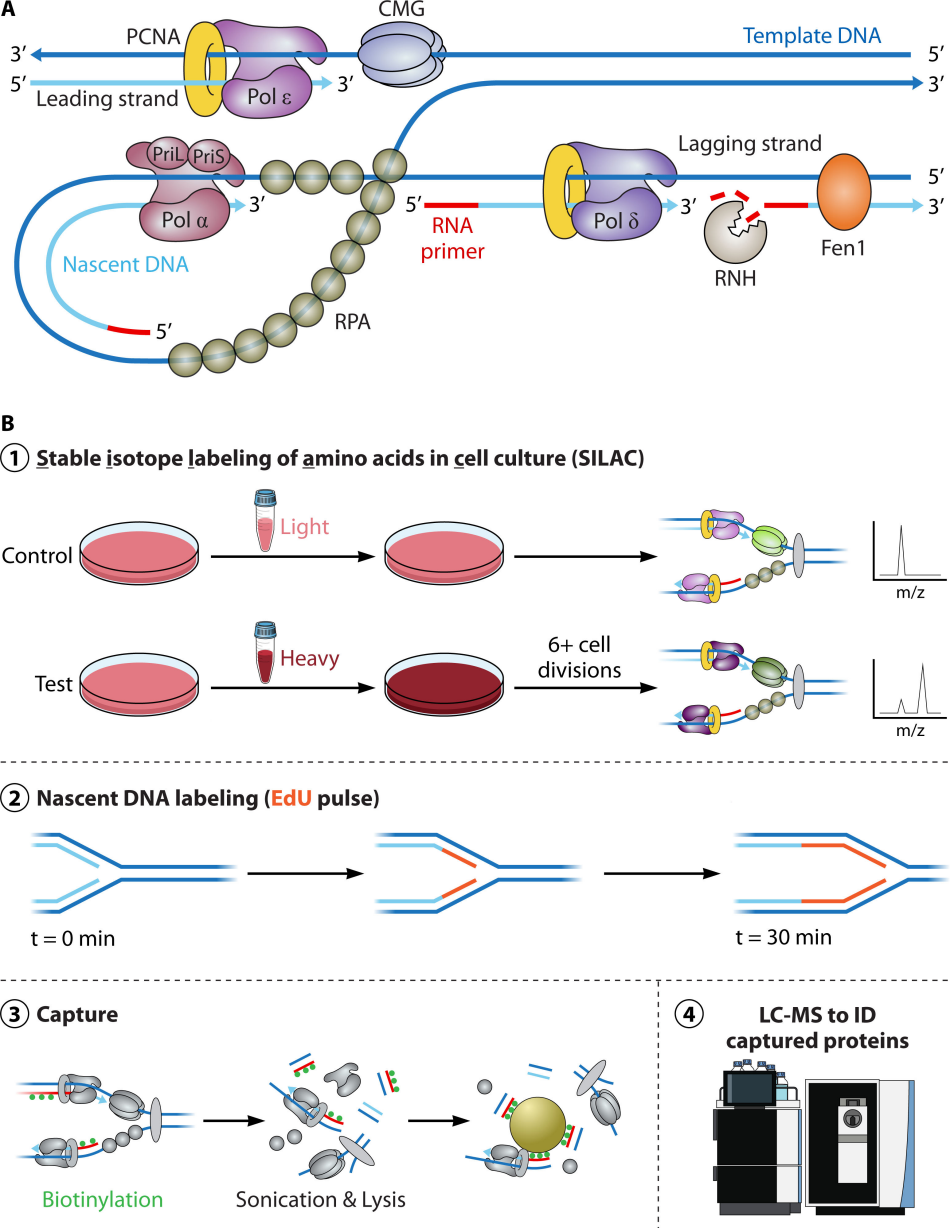
(**A**) Schematic of an unstressed human replication fork and core replication factors. Template DNA (dark blue), nascent DNA (light blue), and RNA (red) are defined by color. (**B**) Overview schematic of isolation of proteins on nascent DNA (iPOND) workflow representing key steps. ① Schematic of stable isotope labeling of amino acids in cell culture (SILAC) labeling of proteins in cells grown for at least six cellular divisions. ② Schematic of temporal EdU incorporation into DNA. Template DNA (dark blue), nascent DNA (light blue), and EdU (red) are indicated by color. ③ Simplified schematic of iPOND methodology including biotinylation (green) of EdU-labeled DNA and affinity capture. ④ iPOND samples are analyzed using liquid chromatography-mass spectrometry (LC-MS).

The host replisome is characterized by core DNA replication factors that travel with the replication fork and broadly consists of the major polymerases, DNA clamp loader proteins, accessory factors, and ssDNA-binding proteins (Fig. 1A). In humans, these consist of the lagging strand polymerase-primase and replication initiation factor Polα, the major lagging strand polymerase Polδ, the major leading strand polymerase Polε, the clamp loader PCNA, the multifactor CMG helicase, and the ssDNA-binding protein RPA that coats DNA and is discontinuously synthesized on the lagging strand. Subsequent studies have further defined the core replisome through *in vitro* biochemical reconstitution systems in bacteria, yeast, and humans (107, 109). Although this handful of proteins will minimally replicate DNA *in vitro*, unbiased proteomic screens defined the replisome in its entirety in human cell lines. The complete replisome (estimated to be ~600 proteins) is far more complex and dynamic than the biochemically-defined core replisome and can be quickly and dramatically altered depending on replication stress and lesions that the replication fork encounters (110). Viruses likely contribute to this dynamic composition as studies posit that HPV may alter and titrate DNA replication repair factors away from human DNA to its own genome (60).

## iPOND AS A TOOL OF DISCOVERY TO DEFINE THE EFFECTS OF VIRAL REPLICATION AND VIRAL ONCOPROTEIN EXPRESSION ON THE COMPOSITION AND FUNCTION OF THE HOST REPLISOME

Loss of replication stress components and complexes, as well as replication stress itself, can rapidly and dynamically remodel the replisome by altering the proteins that interact with actively replicating and mature, chromatinized DNA. iPOND, is a large quantitative, unbiased proteomics-based methodology that, in conjunction with SILAC, has made tremendous strides in recent years to analyze protein dynamics at nascent DNA (111, 112) (Fig. 1B). This method provides opportunities to examine changes in DNA replication and repair machinery (replisomes) during periods of replicative stress, i.e., at active vs stalled replication forks (113), elucidate heretofore unknown mechanisms regulating DNA replication and repair, and define both the normal and dysregulated replisome, i.e., through viral manipulation. It has also been extraordinarily successful in the discovery of both characterized and uncharacterized proteins serving in novel roles during both perturbed and unperturbed replication. For example, a recent proteomic study of replication forks (110) provided a comprehensive catalog of fork-associated proteins and unprecedented mechanistic insights into novel DNA repair mechanisms (114, 115).

Multiple groups have employed iPOND-MS (mass spectrometry) methodologies with great success to understand and define the effects of DNA viruses such as HSV-1, adenovirus, and vaccinia virus on the host replisome, identify mechanisms of viral disruption and commandeering of host proteins, elucidate the composition of viral replisomes, and identify viral restriction factors (116–118). Most virus iPOND studies thus far have been performed in the context of the whole viral life cycle, and to date, none have been performed in HPV nor MCPyV models of infection. Importantly, no studies to date have defined the roles of HPV and MCPyV viral oncoproteins in the holistic remodeling of the human replisome. It is critical that future studies address these open questions. The use of iPOND to analyze HPV E6/E7 or MCPyV T antigens in relevant cell types and/or patient-matched model systems is poised to illuminate tumor-virus oncogene replisome alterations that underlie their oncogenic activities while identifying both similarities and distinctions between viruses.

## CONCLUSIONS AND FUTURE PERSPECTIVES

DNA tumor viruses like HPV and MCPyV have evolved efficient mechanisms to subvert cellular pathways involved in DNA replication and repair, proliferation, signaling, and immune evasion. By studying the dynamic interactions involved in virus-mediated dysregulation of the host replisome, we can improve our understanding of viral pathogenesis and virus-mediated carcinogenesis and uncover fundamental principles of DNA replication and repair. Historically, DNA tumor virus research has facilitated seminal discoveries in molecular biology and cell biology (119). Likewise, we anticipate that several discoveries with broad implications will also emerge from studies focused on HPV and MCPyV modulation of the host replisome.

Although HPV research has been a central focus in the field of viral oncology, strikingly little is known about how HPV infection alters the host replisome, fork stability, alternative DDR pathways, or the role that an HPV-dysregulated host replisome plays in carcinogenesis. Although recent studies strongly support potential oncogenic effects of HPV E6 and E7 during viral replication that augment replication stress through dysregulation of host DDR (60), further investigation is required to determine how this dysregulation affects replisomes. Moreover, whether E6 and E7 have similar effects when the HPV genome is integrated, or whether unrepaired DSBs in host DNA caused by E6 and E7 promote viral genome integration, remain outstanding questions. Future studies comparing cutaneous and mucosal HPVs as well as low-risk and high-risk HPVs will illuminate differences in HPV-mediated host replisome dysregulation across anatomical sites and will likely define changes to the replisome specific to oncogenic HPVs.

Even more questions remain for MCPyV, a virus that has not yet benefitted from decades of research like HPV. For instance, how the MCPyV T antigens mediate host DDR regulation is still coming into focus, and relatively little is known about how these viral proteins alter host replisomes in infected cells. Key experiments are necessary to determine whether full-length LT and the truncated LT found in MCC tumors affect the host replisome in distinct manners. Moreover, whether MCPyV ST and truncated LT alter replisome composition using mechanisms employed by HPV and other DNA tumor viruses, and to what extent, remains unexplored. Previous studies of related polyomaviruses support additional roles of MCPyV LT in manipulating host replication and transcription machinery, as is seen in SV40 through well-characterized LT interactions with replisome factors including RPA (120–122) and the DNA polymerase α-primase complex (123–126), and recently through SV40 LT interactions with ETS1 (127).

Another outstanding question is whether different strains of MCPyV display variable oncogenic potential, akin to HPV. Recent sequencing technology advances have revealed MCPyV strains that exhibit geographic-specific genetic variation in their NCCR (128). Notably, some of the genetic variation observed in MCPyV strains lies within putative transcription factor-binding sites (129), suggesting potential variation in the methods or efficiency by which different MCPyV strains manipulate host replisomes for their own viral propagation. It is also possible that studies of MCPyV will identify shared mechanisms of replisome manipulation by other cutaneous polyomaviruses (130). Finally, future iPOND studies may also help reveal specific alterations to the host replisome by MCPyV T antigens that contribute to the development of MCC, a neuroendocrine cancer, thus potentially providing insight into the genesis of other neuroendocrine cancers.

**Fig 2:**
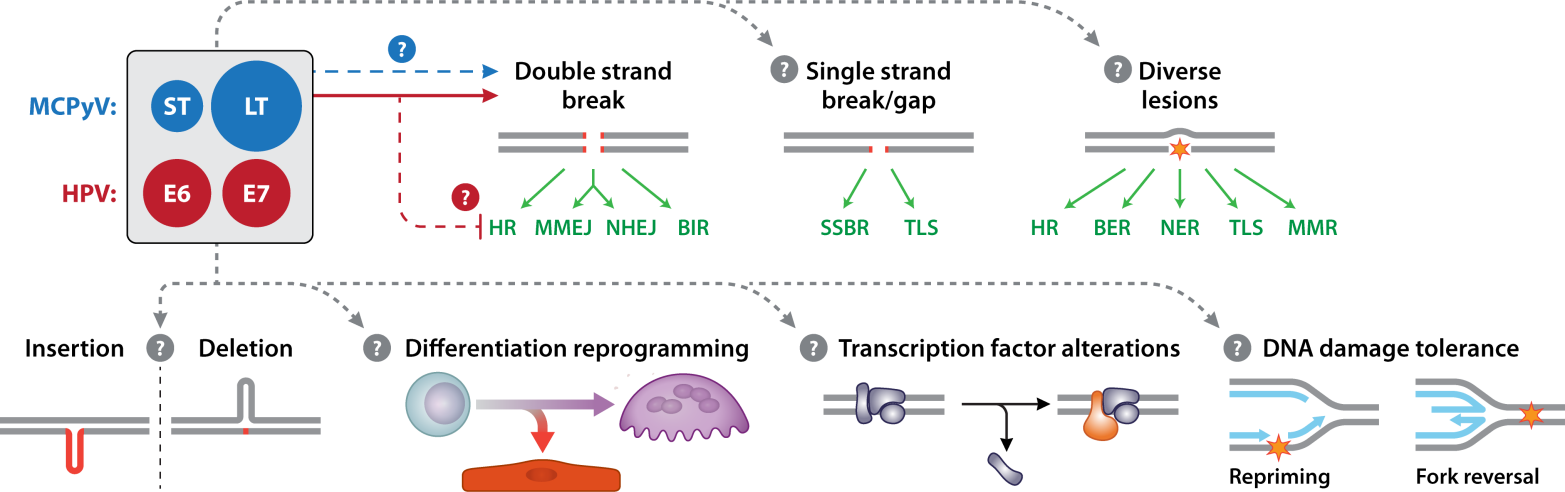
Schematic of possible perturbations to DDR (induced by DSBs, SSBs, INDELS, and diverse lesions including but not limited to base oxidation or deamination, CPDs, and ICLs), replication stress, DNA damage tolerance, cell differentiation/reprogramming, or transcription in host cells expressing HPV oncogenes E6 and E7 or MCPyV ST and LT. HPV and MCPyV oncoproteins with arrows (red and blue, respectively) indicating virus-specific manipulation of DDR in host cells, and arrows indicating unknown/hypothesized manipulation of host cells (gray) are defined by color.

We propose that a deeper investigation into tumor virus-mediated manipulation of host replisomes will illuminate critical regulatory mechanisms governing additional and heretofore uncharacterized DNA repair pathways. Importantly, distinctions must be drawn between the strategies employed by different tumor viruses and their oncogenes—such as HPV and MCPyV—which, while superficially similar, may diverge in how they reprogram host DNA replication and repair dynamics (Fig. 2), contribute to disease, and influence patient outcomes. Elucidating the precise impact of viruses on host replisomes promises powerful insights into the induction of cancer hallmarks and oncogenesis, opening avenues for the design of novel therapeutic interventions against HPV, MCPyV, and other tumor viruses, as well as viruses more broadly.
